# Supplementary material for: Comparison of oral and gut microbiome highlights role of oral bacteria in systemic inflammation in HIV
Source: bioRxiv. 2025 May 16:2025.05.16.654362. Preprint. [Version 1] doi: 10.1101/2025.05.16.654362 (PMC12132388; doi:10.1101/2025.05.16.654362)
Supplement: Supplement 1 [file media-1.pdf]

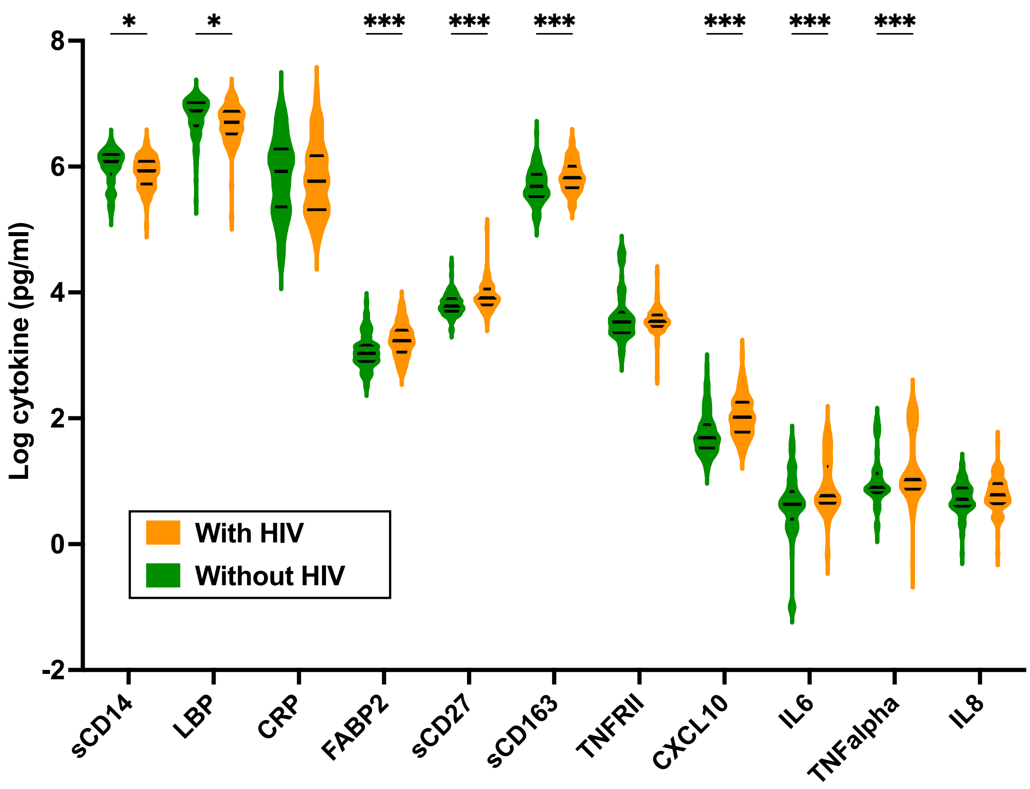

Supplementary Figure 1. Plasma cytokines/biomarkers by HIV groups. Middle lines denote median with top/bottom lines quartiles. *p<0.05; *** p<0.001 by unpaired t-tests.
